# The First Case of Biological Therapy Discontinuation After a Complete Remission Induced by Maintenance Therapy With Adalimumab for Refractory Ulcerative Colitis

**DOI:** 10.14740/jocmr1991w

**Published:** 2014-11-19

**Authors:** Satoshi Tanida, Tsutomu Mizoshita, Keiji Ozeki, Hironobu Tsukamoto, Yoshinori Mori, Eiji Kubota, Hiromi Kataoka, Takeshi Kamiya, Takashi Joh

**Affiliations:** a Department of Gastroenterology and Metabolism, Nagoya City University Graduate School of Medical Sciences, Nagoya, Japan

**Keywords:** Refractory ulcerative colitis, Adalimumab, Biologic discontinuation, Complete remission

## Abstract

A 43-year-old woman, diagnosed with ulcerative colitis (UC) at age of 30, received outpatient treatment with corticosteroids. However, flare-up occurred, and adalimumab (ADA) treatment commenced in July 2009. A complete remission with mucosal healing was achieved by 32 weeks after initiation of ADA therapy. Because of progressive skin eruptions, ADA maintenance was discontinued at 124 weeks. Regardless, complete remission with mucosal healing was maintained until 176 weeks. We concluded that ADA is an effective therapy to achieve a complete remission in a patient with steroid-refractory UC, and that long-term complete remission may be an important indication to discontinue biological therapy.

## Introduction

Ulcerative colitis (UC) is characterized by mucosal ulceration, rectal bleeding, diarrhea, and abdominal pain. Studies have described the efficacy and safety of adalimumab (ADA) and infliximab (IFX) for treatment of patients with moderate-to-severe UC who failed to achieve clinical remission or to respond to conventional therapy consisting of corticosteroids, azathioprine, and/or aminosalicylic acid (5-ASA) [1-3]. A statement from the World Congress of Gastroenterology suggests that biologics therapy can be withdrawn in cases of complete mucosal healing in patients with Crohn’s disease (CD) [4]. However, there is currently a lack of evidence supporting the discontinuation of anti-tumor necrosis factor (TNF)-α antibodies for UC patients in complete remission. To our best knowledge, there is also no case report of discontinuation of ADA after institution of scheduled maintenance. This report describes the first case of a patient with refractory UC in which biologic therapy was terminated after achieving complete remission in response to scheduled maintenance therapy with ADA.

## Case Report

**Figure 1:**
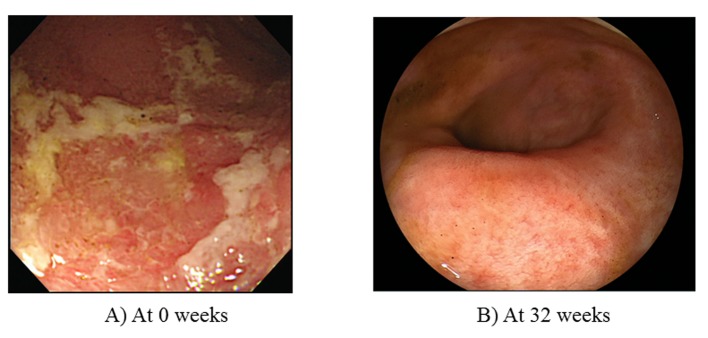
Colonoscopy before (at 0 week) and at 32 weeks after induction and maintenance therapy with ADA (160/80/40 mg every other week). (A) The first colonoscopy reveals some ulcers with erythematous and edematous changes within the mucosa of the sigmoid and rectum. Mayo endoscopy subscore, 3 points. (B) Colonoscopy reveals mucosal healing with scars within the mucosa of the sigmoid colon following 32 weeks of treatment.

A 43-year-old woman, diagnosed with total UC at age 30, received outpatient treatment with corticosteroids. In April 2009, she experienced a flare-up of colitis and required daily oral sulfasalazine (4,500 mg/day) and 10 mg of prednisolone. However, these drugs could not induce clinical remission. The Mayo score, measured after 2 weeks to evaluate disease severity was 9 points (stool frequency, 3 points; rectal bleeding, 1 point; physician’s global assessment, 2 points; endoscopy subscore, 3 points (Fig. 1A)), indicating moderately active UC. Laboratory investigations revealed a white blood cell (WBC) count of 7,240 μ/L, a red blood cell (RBC) count of 379 × 10^4^/μL, a hemoglobin (Hb) of 13.1 g/dL, a total protein (TP) of 6.9 g/dL, and a C-reactive protein (CRP) of 0.11 mg/dL. ADA induction therapy (160/80 mg) was initiated in July 2009 (at 0 week), followed by 40 mg at 4 weeks and every other week thereafter. At 32 weeks, complete remission with mucosal healing was achieved (Mayo score, 1 point; endoscopy subscore, 0 (Fig. 1B)). Corticosteroids were tapered off by 20 weeks on the basis of a good response to ADA treatment.

At 52 weeks, the patient developed skin eruptions at the injection site that progressed to involve her arm and neck. Skin eruptions receded soon after administration of anti-allergic agents. However, skin eruptions persisted at the injection site in her arm. Subsequently, skin eruptions again progressed to extend from her arm to her eyelids, and erythematous changes were finally seen in her conjunctiva at 124 weeks, so ADA was immediately discontinued. Skin eruptions gradually disappeared in a couple of weeks. She has not experienced allergic skin eruptions since a definite diagnosis of UC was made, and skin eruptions were improved soon after ADA was discontinued. This clinical course suggested that she was considered to be allergic for ADA, but not to have a complication of UC. Complete remission with mucosal healing (endoscopy subscore, 0) was noted at that time and was sustained beyond 176 weeks without additional therapeutic agents for UC treatment (Table 1).

**Table 1:** The Clinical Course of Mayo Scores and Eruption Severity With Concomitant Drugs’ Doses Including SASP, Prednisolone, and ADA

Times from ADA start (weeks)	-2	0	2	4	8	32	52	78	104	124	130	176
SASP (mg)	4,500	4,500	4,500	4,500	4,500	4,500	4,500	4,500	4,500	4,500	4,500	4,500
Corticoteroids (mg)	10	10	10	10	10	0	0	0	0	0	0	0
ADA injections (mg)	-	160	80	40	40	40	40	40	40	Discontinued	-	-
Mayo scores	9	6#			6	1	1	1	1	1#	1	1
Bowel movements (points)	3	3			3	1	1	1	1	1	1	1
Anal bleeding (points)	1	1			1	0	0	0	0	0	0	0
Endoscopic score (points)	3				1	0	0	0	0		0	0
Adverse events												
Skin eruptions	-	-			-	-	+	++	+	+++	-	-

#Partial Mayo scores without endoscopy subscore. -: none; +: mild; ++: moderate; +++: severe.

## Discussion

In the clinical setting, there are little data to guide optimal discontinuation of ADA after institution of scheduled maintenance. The present report described the first case of a patient who achieved biologic discontinuation after a complete remission induced by induction/maintenance therapy with ADA for the treatment of refractory UC.

Anti-TNF-α antibodies, such as IFX and ADA, are effective therapies that can lead to sustained clinical remission in patients with moderate-to-severe active UC [1, 2]. Treatment with anti-TNF-α antibodies has revolutionized the treatment of refractory UC. Clinical remission or mucosal healing is currently a realistic goal for most patients. Many studies have assessed strategies for discontinuation of biologic therapy in patients with CD and rheumatoid arthritis.

One prospective cohort study involved 115 patients with CD who were treated for at least 1 year with scheduled IFX and an antimetabolite and had been in corticosteroid-free remission for at least 6 months, and who subsequently received IFX discontinuation during a study of IFX discontinuation in CD patients in stable remission on combined therapy with immunosuppressors (STORI) trial. The study showed that risk factors for relapse involved the absence of surgical resection, male sex, leukocyte counts > 6.0 × 10^9^/L, levels of Hb ≤ 145 g/L, and CRP ≥ 5.0 mg/L, and fecal calprotectin ≥ 300 μg/g, and that patients with no more than two of these risk factors had a remarkably low risk of relapse [5].

An open-label pilot study of the feasibility of discontinuation of ADA in rheumatoid arthritis patients in stable clinical remission demonstrated that 20% of patients receiving ADA treatment achieved biologic discontinuation for the first 28 weeks and that failure of sustained low disease activity index (DAS28) of rheumatoid arthritis failed to discontinue biologics [6]. On the other hand, a systematic review evaluating biologic discontinuation in patients with rheumatoid arthritis could not show any significant factors relevant to biologic discontinuation or failure of biologic discontinuation, as collective studies reviewed were relatively small [7]. Thus, although the feasibility factors of biologic discontinuation remain to be defined, long-standing clinical remission or remarkably low disease activity may be an important indication for biologic discontinuation.

Logistic regression analysis in a prospective observational study evaluating predictors of relapse in patients with UC in remission after 1 year of IFX treatment showed that only previous biological therapy (repeated treatment with IFX) was associated with the necessity of restarting IFX therapy and that none of the analyzed demographic and clinical parameters (including sex, smoking, steroid/thiopurine therapy, history of colon surgery, appendectomy, dose intensification, and CRP level) was associated with the necessity of restarting IFX therapy. In addition, restart of IFX therapy seems to be more common in patients without mucosal healing at the time of IFX discontinuation, although there was no significant correlation between mucosal healing and the frequency of, nor time to, clinical relapse [8].

Two-year complete remission with mucosal healing (Mayo endoscopy subscore, 0) was maintained in patients with UC who were treated for about 2.5 years with scheduled ADA and also in the present case where the patient had been in corticosteroid-free remission for 2 years. Therefore, mucosal healing was considered to be an indication for successful biologic discontinuation. Because of adverse events, ADA was discontinued in the present case.

On the other hand, a recent retrospective observational study assessing prognostic role of mucosal healing in 22 Hungarian patients with UC after 1 year of biological therapy concluded that mucosal healing did not predict sustained clinical remission in patients in whom the biological therapy had been stopped based on the result that all of five UC patients achieving both mucosal healing and clinical remission received retreatment of biological therapy within 1 year [9]. Further prospective study is needed to determine whether long-term complete remission with mucosal healing enables UC patients to discontinue biologics.

In conclusion, ADA is an effective therapy to achieve a complete remission in patients with refractory UC and long-term complete remission may be an important indication of discontinuing biological therapy.

## Abbreviations

UC: ulcerative colitis
CD: Crohn’s disease
CRP: C-reactive protein
ADA: adalimumab
IFX: infliximab
SASP: sulfasalazine
5-ASA: 5-aminosalicylic acid
DAS: disease activity score
